# Intermembrane crosstalk drives inner-membrane protein organization in *Escherichia coli*

**DOI:** 10.1038/s41467-018-03521-4

**Published:** 2018-03-14

**Authors:** Patrice Rassam, Kathleen R. Long, Renata Kaminska, David J. Williams, Grigorios Papadakos, Christoph G. Baumann, Colin Kleanthous

**Affiliations:** 1 Department of Biochemistry, University of Oxford, South Parks Road, Oxford, OX1 3QU UK; 2 Department of Biology, University of York, York, YO10 5DD UK; 3 Present Address: Laboratoire de Bioimagerie et Pathologie, UMR 7021, CNRS, Université de Strasbourg, Faculté de pharmacie, 74 Route du Rhin, 67401 Illkirch, France; 4 Present Address: Wellcome Trust Sanger Institute, Wellcome Genome Campus, Cambridge, CB10 1SA UK; 5 Present Address: Division of Molecular Microbiology, School of Life Sciences, University of Dundee, Dow Street, Dundee, DD1 5EH UK; 6 Present Address: Division of Neurobiology, The Roslin Institute, The University of Edinburgh, Easter Bush, Midlothian, EH25 9RG UK

## Abstract

Gram-negative bacteria depend on energised protein complexes that connect the two membranes of the cell envelope. However, β-barrel outer-membrane proteins (OMPs) and α-helical inner-membrane proteins (IMPs) display quite different organisation. OMPs cluster into islands that restrict their lateral mobility, while IMPs generally diffuse throughout the cell. Here, using live cell imaging of *Escherichia coli*, we demonstrate that when transient, energy-dependent transmembrane connections are formed, IMPs become subjugated by the inherent organisation of OMPs and that such connections impact IMP function. We show that while establishing a translocon for import, the colicin ColE9 sequesters the IMPs of the proton motive force (PMF)-linked Tol-Pal complex into islands mirroring those of colicin-bound OMPs. Through this imposed organisation, the bacteriocin subverts the outer-membrane stabilising role of Tol-Pal, blocking its recruitment to cell division sites and slowing membrane constriction. The ordering of IMPs by OMPs via an energised inter-membrane bridge represents an emerging functional paradigm in cell envelope biology.

## Abstract (web summary)

Outer membrane proteins (OMPs) in Gram-negative bacteria have restricted lateral mobility. Here, Rassam et al. show that the bacteriocin ColE9, via its interactions with OMPs, imposes this restricted mobility on the inner membrane proteins of the Tol-Pal complex.

## Introduction

The importance of the cell envelope in Gram-negative bacteria is emphasised by the large number of genes devoted to its functional integrity—for *Escherichia coli*, this is over a quarter of its genome—and by the many antibiotics that target it^1^. Coordination between the outer and inner membranes, which are ~25 nm apart^2^, is thought to be integral to cell envelope biology, but how this occurs and to what extent is not known. Connections between the outer and inner membranes can be permanent or transient in nature. Permanent structures include type III and type VI secretion machines that reside in both membranes and deliver virulence factors into host cells^3,4^. Transient connections require an energy source, typically the PMF, to promote contact between proteins in the two membranes. Such energised protein complexes serve many functions, including gliding motility^5^, antibiotic efflux^6^, lipopolysaccharide secretion^7^ and nutrient import^8^, as well as being parasitized by bacteriocins^9^ and bacteriophages^10^ to promote their entry into bacterial cells.

**Fig. 1:**
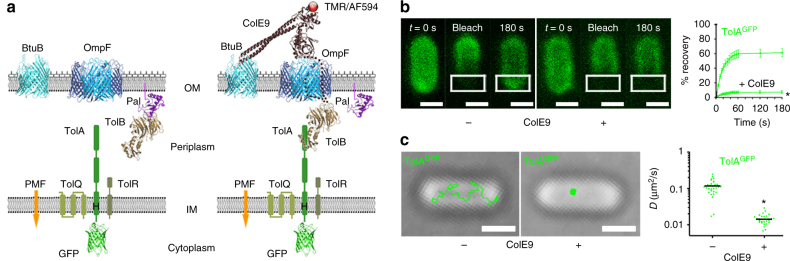
OMP-bound ColE9 reduces the lateral mobility of TolA in the IM. **a** Left-hand panel, central components of the Tol-Pal complex (TolQ, TolR and TolA in the IM, TolB in the periplasm and the lipoprotein Pal in the OM) and the OMPs BtuB and OmpF. ColE9 exploits all these proteins during its import with the exception of Pal. TolA was tagged with GFP at its N-terminus while TolQ was fused to mCherry at its C-terminus (not shown). Also shown is the location of a histidine residue (H22) in the transmembrane helix of TolA that is critical for coupling to the PMF via TolQ and TolR^36^. Right-hand panel, binding of fluorescently labelled nuclease bacteriocin ColE9 to BtuB/OmpF recruits TolB, which in turn binds TolA in the IM^43^ and establishes a protein bridge across the cell envelope. The colicin contains an internal disulphide bond that blocks import. Figure adapted from Rassam, P., Copeland, N.A., Birkholz, O., Tóth, C., Chavent, M., Duncan, A.L., Cross, S.J., Housden, N.G., Kaminska, R., Seger, U., Quinn, D.M., Garrod, T.J., Sansom, M.S.P., Piehler, J., Baumann, C.G. & Kleanthous, C. Supramolecular assemblies underpin turnover of outer-membrane proteins in bacteria. *Nature* **523**, 333–336 (2015). **b** Confocal FRAP data for *E*. *coli* JW0729/pNP4, pRP5 cells expressing GFP-TolA (not induced) and BtuB (induced), respectively, in the absence (−) and in the presence (+) of ColE9 (300 nM) and the corresponding recovery curves (averages from 30 cells). Asterisk, *p* < 0.01 from a Student’s *t* test. **c** Single-molecule TIRFM tracking data of GFP-TolA in the IM before and after ColE9 forms its transenvelope complex showing how OMP-bound colicin restricts the lateral mobility of TolA. Measurements of the TolA diffusion coefficient from mean square displacement plots of single-molecule trajectories are shown alongside, where *n* = 30. Those of TolA in the presence of ColE9 are typical of OMPs such as BtuB^16^. Asterisk, *p* < 0.001 from a Mann–Whitney *U*-test. Scale bars, 1 μm

We investigated inter-membrane coupling across the cell envelope of *E*. *coli* in the context of Tol-Pal recruitment by the OMP-bound bacteriocin colicin E9 (ColE9). The Tol-Pal system, which is comprised of the OM lipoprotein Pal, TolB in the periplasm and the TolQ/TolA/TolR complex in the IM, is found in most Gram-negative bacteria and contributes to virulence in many pathogens^11^. The system is recruited late to the cell division apparatus by the divisome protein FtsN, where it stabilises the OM by a PMF-driven mechanism that has yet to be elucidated^12^. Many bacteriocins (known as group A colicins) parasitise the Tol-Pal assembly in *E*. *coli* presumably because the system spans the cell envelope and provides an energy source for import^13^. Access to this energy source however requires formation of a translocon in which the OM and IM become connected^14^. In the case of ColE9, previous work has shown that its translocon involves the colicin simultaneously contacting the vitamin B_12_ transporter, BtuB, and the porin, OmpF, in the OM, and the TolQ/TolA/TolR assembly in the IM via the periplasmic protein TolB (Fig. 1a)^9^. Translocon formation catalyses import of the colicin’s cytotoxic endonuclease (DNase) domain, ultimately resulting in death of the cell due to cleavage of the bacterial chromosome^15^.

The lateral mobility of OMPs is restricted due to their sequestration within OMP islands^16^. By contrast, IMPs are generally free to diffuse throughout the cell^17,18^. Given these marked differences and the importance of inter-membrane systems to bacteria, the present work set out to establish how transient, energy-dependent contact between OMPs and IMPs across the cell envelope impacts IMP mobility, organisation, and function. We find that when ColE9 establishes its translocon complex, the IMPs of the Tol-Pal assembly take on the mobility and organisation of the OMPs to which the colicin is bound. This OMP-imposed organisation is dependent on the PMF and subverts the native function of the Tol-Pal assembly.

## Results

### OMP-bound ColE9 restricts IMP lateral mobility

We site-specifically coupled fluorophores, tetramethyl rhodamine (TMR) or Alexa Fluor 594 (AF594), to the C-terminal DNase domain of ColE9, using a variant compromised for OM import but not translocon assembly due to an intramolecular disulphide bond within its receptor-binding domain^16^. The connection of the OMP-bound ColE9 to the IMP complex of TolQ/TolA/TolR was visualised using either GFP-TolA or TolQ-mCherry (Fig. 1a, Supplementary Figure 1a and b; see Methods for details)^12,19^. We initially used fluorescence recovery after photobleaching (FRAP) to determine the impact of ColE9 forming its translocon on IMP mobility. ColE9^TMR^-labelled BtuB/OmpF showed no fluorescence recovery, as observed previously for these and other OMPs (Supplementary Figure 2a)^20^. By contrast, both GFP-TolA and TolQ-mCherry showed rapid recovery of fluorescence, indicative of mobility in the IM (Supplementary Figures 2a, 3a and c). OmpF is one of the most abundant proteins in the OM (~10^5^ copies per cell) while BtuB has a low copy number similar to that of Tol proteins (~10^3^ copies per cell)^21^. We hypothesised that the levels of BtuB relative to OmpF might mask any changes to IMP mobility. We therefore repeated the FRAP experiments in cells where BtuB levels were raised fivefold. Now, both GFP-TolA and TolQ-mCherry showed no fluorescence recovery in FRAP experiments when ColE9 formed its translocon complex (Fig. 1b; Supplementary Figures 2b–d and 3a and c), mirroring the behaviour of the colicin-labelled OMPs. Hence, OMP-bound ColE9^TMR^ is able to influence the mobility of TolA and TolQ in the IM even though it only makes contact with these components of the Tol assembly indirectly through TolB in the periplasm.

We next sought to probe the OMP–IMP link and the level of IMP restriction, focusing on GFP-TolA. Disruption of the inter-membrane connection by proteolysing surface-bound ColE9, deleting the *tolB* gene or TolB-binding sequences in the colicin (Δ1-83 ColE9) all reinstated fluorescence recovery in GFP-TolA FRAP experiments (Supplementary Figure 4). Finally, total internal reflection fluorescence microscopy (TIRFM) measurements of single GFP-TolA molecules indicated that TolA in the presence of ColE9 displayed highly restricted lateral motion, whereas TolA in the absence of colicin exhibited unrestricted motion typical of IMPs (Fig. 1c)^20^. Addition of trypsin, use of Δ1-83 ColE9 or deletion of *tolB* all reversed these effects (Supplementary Figure 5). Our results demonstrate that the normally free-diffusing IMP TolA exhibits lateral mobilities and diffusion properties similar to those of the OMPs to which it becomes coupled by a bacteriocin-mediated transenvelope bridge.

### Tol-Pal IMP clusters mirror those of OMP-bound ColE9

**Fig. 2:**
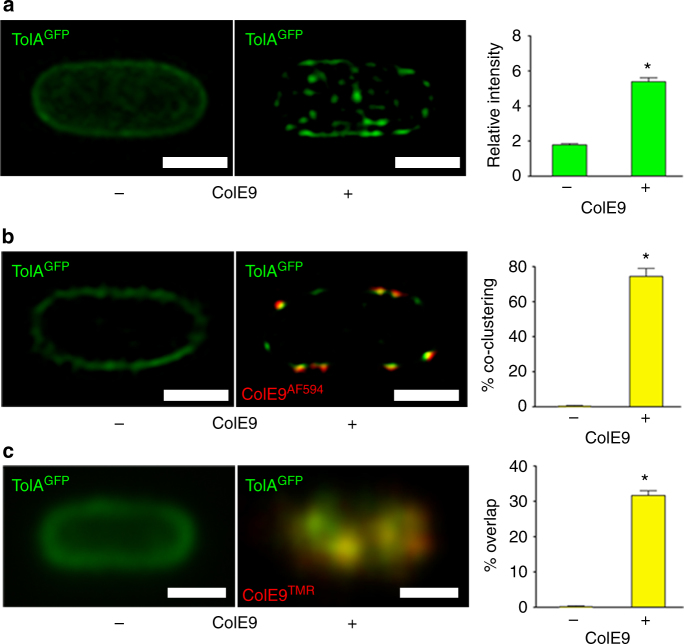
Bacteriocin-induced clustering of TolA in the IM mirrors that of OMPs. **a** Typical 3D-SIM images of *E*. *coli* JW0729/pNP4, pRP5 cells expressing GFP-TolA (not induced) and BtuB (induced), respectively, showing how formation of a transenvelope complex by ColE9 induces clustering of TolA in the IM. **b** 2D-SIM *z*-slice showing significant co-clustering (yellow fluorescence) of GFP-TolA and ColE9^AF594^ in the IM and OM, respectively, along with quantitation of the degree of co-clustering in the accompanying histogram. **c** TIRFM data showing co-localisation of GFP-TolA clusters in the IM and OMP-bound ColE9^TMR^ islands (yellow fluorescence) along with quantitation of the degree of overlap in the accompanying histogram. Scale bars, 1 μm. Asterisk, *p* < 0.01 from a Student’s *t* test for *n* = 30 cells in each of the histograms shown. See Methods for details

The restricted lateral mobility of OMPs in the *E*. *coli* OM is due to their clustering within OMP islands^16,20^. We therefore investigated whether the restricted mobility imposed on GFP-TolA and TolQ-mCherry by OMP-bound ColE9^TMR^ was indicative of clustering. Three-dimensional (3D)-structured illumination microscopy (3D-SIM) showed that TolA is distributed throughout the cell but that addition of ColE9-induced clustering in the IM (Fig. 2a; Supplementary Figures 3b and d; Supplementary Movies 1 and 2), similar to that previously reported for BtuB-labelled ColE9^TMR^ in the OM^16^. By taking single *z*-slices through the central regions of GFP-TolA cells (2D-SIM) and scoring the degree of green/red fluorescence pixel overlap (thereby extending the nominal ~150 nm resolution limit of these experiments), we were able to demonstrate that ColE9^TMR^ induced significant co-clustering of OMPs and IMPs (Fig. 2b) (see Methods for details). Co-clustering was also observed in TIRFM experiments for both GFP-TolA and TolQ-mCherry (Fig. 2c and Supplementary Figure 3d). Disruption of the transenvelope bridge by trypsin treatment or by deletion of *tolB* abolished the co-clustering of OMPs and IMPs (Supplementary Figure 6a–c). Finally, by impregnating agar pads with ColE9 onto which cells were laid, we could visualise the emergence of clusters in the IM (Supplementary Figure 6d). We conclude that the ColE9-induced clustering of TolA and TolQ in the IM mirrors that of OMPs in OMP islands, which are the result of promiscuous protein–protein interactions between β-barrel proteins^20^.

### IMP-induced clustering by OMP-bound ColE9 requires the PMF

**Fig. 3:**
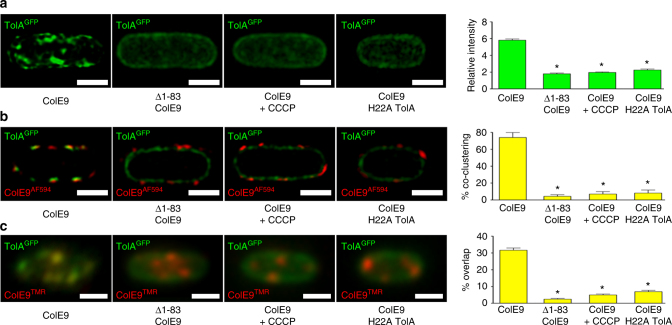
ColE9/OMP-induced organisation of TolA in the IM requires the PMF. Panels show JW0729/pNP4, pRP5 cells expressing GFP-TolA (not induced) and BtuB (induced), respectively, bound with ColE9^AF594^ and visualised by (**a**) 3D-SIM (GFP fluorescence only), (**b**) 2D-SIM *z*-slices (GFP-TolA and ColE9^AF594^ fluorescence) and (**c**) TIRFM data (GFP-TolA and ColE9^TMR^ fluorescence). First column, addition of ColE9^AF594^ alone. Second column, addition of Δ1-83 ColE9^TMR/AF594^ in which the connection to TolA is broken, resulting in the loss of both GFP-TolA clustering and GFP-TolA-ColE9^TMR/AF594^ co-localisation. Third column, addition of CCCP (0.1 mM) destroys the PMF yielding a similar effect to that of the Δ1-83 ColE9^TMR/AF594^ mutant. Fourth column, addition of ColE9^TMR/AF594^ to *E*. *coli* JW0729/pREN88, pRP5 cells expressing GFP-H22A TolA (uninduced) and BtuB (induced), respectively. Scale bars, 1 μm. Asterisk, *p* < 0.01 from a Student’s *t* test for *n* = 30 cells shown in each set of data in the accompanying histograms

Transient connections between the Tol-Pal complex and the OM are known to involve the PMF^22,23^. 3D-SIM, 2D-SIM, and TIRFM experiments demonstrated that disruption of the PMF-coupling mechanism, either by addition of the protonophore CCCP (before or after addition of colicin; Supplementary Figures 5 and 6) or through mutation to alanine of an essential TolA transmembrane histidine residue (H22A)^23^, all abolished the ColE9^TMR^-induced clustering behaviour of GFP-TolA (Fig. 3). Disruption of PMF coupling also reversed the effects of ColE9 on the diffusion coefficient of GFP-TolA (Supplementary Figure 5). Hence, the ability of colicin-bound OMPs to influence the lateral mobility and organisation of TolA in the IM is dependent on the PMF, which in turn implies the entire TolQ/TolA/TolR PMF-harnessing complex is captured. The requirement for the PMF likely reflects the transient nature of the interactions between Tol-Pal components in the two membranes^22^ and the extension of TolA through the periplasm to contact ColE9-bound TolB close to the OM^9^.

### OMP-bound ColE9 subverts the native function of Tol-Pal

**Fig. 4:**
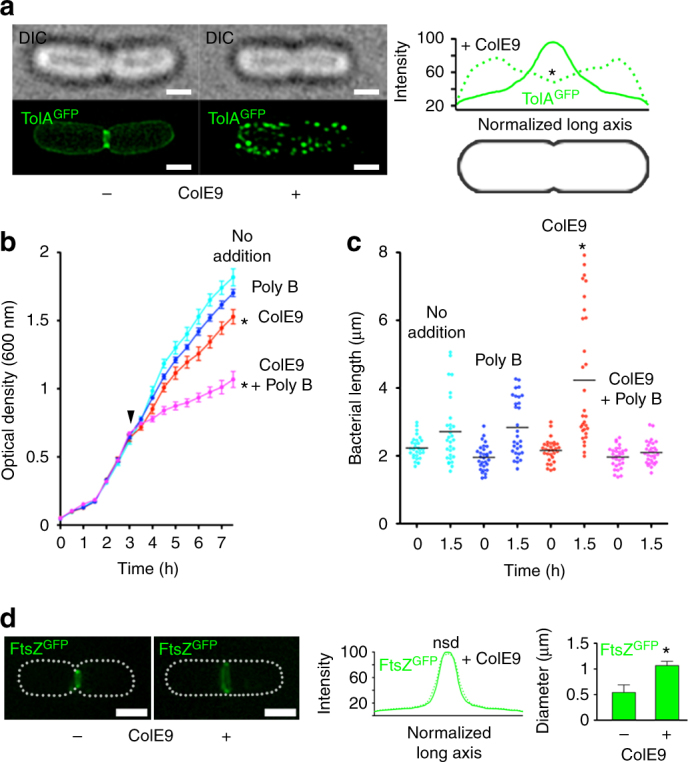
OMP-bound ColE9 sequesters TolA away from the cell division apparatus and destabilises the OM. **a** Representative DIC and 3D-SIM images of dividing *E*. *coli* JW0729/pNP4, pRP5 cells expressing GFP-TolA (not induced) and BtuB (induced), respectively, in the absence (−) and presence (+) of ColE9, respectively. The differential distribution of GFP-TolA across cells, displayed graphically on the right-hand side, shows how formation of the colicin-mediated transenvelope bridge pushes TolA towards the poles and diminishes recruitment to the divisome. Asterisk, *p* < 0.001 from a Mann–Witney *U*-test from 30 cells. **b** Growth curves for JW0729/pNP4, pRP5 cells expressing GFP-TolA (not induced) and BtuB (induced), respectively, in the absence or presence of polymyxin B (144 nM), ColE9 or a mixture of polymyxin B and ColE9. Results shown are for M9 minimal media but the same results were obtained for rich media (Methods). Cells are able to grow at the concentration of polymyxin B used in these experiments unless ColE9 is included, which indicates that the transenvelope bridge formed by the colicin increases the permeability of the OM. Asterisk, *p* < 0.01 from a Student’s *t* test. **c** Distribution of cell length for JW0729/pNP4, pRP5 cells expressing GFP-TolA (not induced) and BtuB (induced), respectively, imaged by differential interference contrast (DIC) microscopy at the beginning of the growth experiment (0 h) and after growth (1.5 h) on agar pads, in the absence or presence of polymyxin B, ColE9 and a ColE9/polymyxin B mixture. ColE9-induced capture of TolA results in significant elongation of cells. Asterisk, *p* < 0.001 from a Mann–Witney *U*-test. **d** Representative 3D-SIM images of FtsZ-GFP and BtuB expressing *E*. *coli* PB199/pRP5 cells grown in agar pads in the absence (−) and presence (+), respectively, of ColE9 and imaged at the same time point (45 min). Normalised fluorescence distributions show that the location of the *Z*-ring is unaltered, but the diameter of the ring increases significantly suggesting ring closure is delayed and explains the cell elongation phenotype in **c**. Scale bars, 1 μm^44^

The Tol-Pal system of *E*. *coli* is recruited to the divisome late during cell division to stabilise the OM during septation^12^. Consistent with this recruitment, 3D-SIM experiments showed GFP-TolA formed a septal ring similar to that of other divisome proteins (Fig. 4a; Supplementary Movie 3)^24^. The clustering of GFP-TolA induced by OMP-bound ColE9 largely abrogated recruitment of TolA to the divisome, an effect that was reversed by trypsin treatment, disruption of translocon assembly or by uncoupling Tol-Pal from the PMF (Fig. 4a, Supplementary Figure 7 and Supplementary Movie 4). While the clustering of GFP-TolA by OMP-bound ColE9 caused only a mild growth defect closer inspection of cell dimensions showed a significant increase in the average length of cells (Fig. 4b, c). Similar defects are seen for a *tolA* deletion strain (Supplementary Figure 8), consistent with the Tol-Pal complex being required for appropriate OM invagination during septation^12^. Indeed, 3D-SIM of cells expressing GFP-labelled FtsZ, the tubulin homologue that coordinates septation in *E*. *coli*^25^, showed that ColE9 capture of TolA delayed closure of the constricting *Z*-ring explaining why cells become elongated (Fig. 4d). We also found that clustering of TolA due to its transenvelope bridge to OMP-bound ColE9 significantly increased susceptibility of *E*. *coli* towards polymyxin B (Fig. 4b, d), a phenotype essentially identical to that of the *tolA* deletion, and EDTA, both of which disrupt the integrity of the OM (Supplementary Figure 8). Control experiments demonstrated that the SOS-response, which is activated by ColE9 following entry to the cytosol^26^, was not induced in these experiments confirming that ColE9 remained at the OM (Supplementary Figure 8e). These data suggest that recruitment of TolA by OMP-bound ColE9 subverts its native function at cell division sites leading to destabilization of the OM. We suggest that such membrane destabilization could open a path to the periplasm for the colicin as well as other group A colicins such as ColA, ColK, ColN, ColS4, and Colicins E2-E9 and, as well as filamentous bacteriophages, all of which exploit the Tol-Pal system^27^.

The imposition of order on IMPs by self-organised OMPs through transient, energised protein bridges is likely to be a principle found more generally in Gram-negative bacteria, as in the functioning of multidrug efflux pumps. Bergmiller et al.^6^ have shown that the PMF-driven, inner-membrane ABC transporter AcrB accumulates at the old pole of *E*. *coli* cells due to TolC, the OMP tunnel through which drugs are ejected. Other systems where the functional organisation of IMPs could be influenced by the OMPs to which they are energetically-coupled include the LPS secretion pathway^7^, the import of nutrients through TonB-dependent transporters^28^ and gliding motility in bacteria^5^. A similar transenvelope organising principle may also be at work in mitochondria and chloroplasts. These organelles, which evolved from endosymbiotic Gram-negative bacteria^29^, have β-barrel OMPs that are known to cluster^30^ and energised links between the two membranes^31^.

## Methods

### Plasmids and bacterial strains

Plasmid pNP4 expressing GFP-TolA was kindly provided by Pete de Boer^12^. Plasmid pREN88 (expressing GFP-TolA H22A) was generated from pNP4, using QuikChange mutagenesis. PCR and isothermal in vitro recombination^32^ was used to replace the ampicillin resistance gene of pNGH15 (pBAD/Myc-His B with *btub* cloned at the NcoI/XhoI sites) with the chloramphenicol resistance gene from pACYC184 to give the plasmid pRP5. All PCR primers used are shown in Supplementary Table 1. All strains used are listed in Supplementary Table 2. The reference Keio strain against which all comparisons were made was *E*. *coli* BW25113^33^. The deletion strain BW25113 *ΔtolA* (JW0729) was also obtained from the Keio collection.

The double-deletion strain GPCK5 (*ΔtolAtolB)* is a derivative of BW25113. It was constructed by λ-Red recombination based on the gene-doctoring protocol^34^ with the following modifications. Plasmid pGP71 was constructed by introducing homology regions upstream and downstream the kanamycin acyltransferase cassette (Kan^r^) of the pDOC-K^34^ plasmid while maintaining the P_B_ promoter of the *tol* operon. A homology region of 300 bp upstream *tolA* was PCR-amplified from BW25113 genomic DNA and ligated in the EcoRI/AgeI digest of pDOC-K giving pGP70. A homology region of 300 bp downstream *tolB* and P_B_ promoter region were also PCR-amplified and joined together by PCR to prepare a SacI/SpeI fragment, which was ligated to the corresponding digest of pGP70. The sequence of the resulting insert in pGP71 was: EcoRI-300 bp upstream *tolA*-AgeI-Kan^r^-SacI- P_B_ promoter-300 bp downstream *tolB*-SpeI.

BW25113 cells carrying the pACBSCE^34^ vector were grown in LB media, and made electro-competent after two washes with ice-cold sterile deionized-distilled water. The insert of pGP71 was EcoRI/SpeI excised from the vector, gel purified (Qiagen) and 1 μg was electroporated in the above cells at 1350 V, 10 μF, 600 Ω using a 1 cm cuvette (Bio-Rad Gene Pulser). The cells were recovered by growth in LB media without antibiotics and plated on plates supplemented with 50 μg/ml kanamycin. The gene deletions were confirmed by colony PCR and DNA sequencing.

A similar method was used to engineer RKCK5, a *gfp-tolA* derivative of BW25113. Site-directed mutagenesis was used to abolish an internal *gfp* MunI restriction site in pNP4. This enabled cloning of PCR-amplified *gfp-tolA* between AgeI and MunI of pGP70. The resulting pREN98 was subjected to site-directed mutagenesis to delete AgeI restriction site between the beginning of *gfp* and the homology region of 300 bp upstream of *tolA*. The 300 bp region downstream of *tolA* was PCR-amplified from BW25113 genomic DNA and inserted between SacI and SpeI sites of the same construct. The sequence of the resulting insert in pREN100 was: EcoRI-300 bp upstream *tolA-gfp-tolA-*MunI-Kan^r^-SacI-300 bp downstream *tolA*-SpeI. The insert was then EcoRI/SpeI excised from the vector and electroporated into BW25113/pACBSCE cells. The cells were recovered by growth in LB media without antibiotics and plated on plates supplemented with 50 μg/ml kanamycin. The gene insertions were confirmed by colony PCR and DNA sequencing. The kanamycin cassette was then excised from the chromosome, as described previously^35^. The deletion was confirmed by colony PCR and DNA sequencing.

JW0729 and GPCK5 strains were both transformed with pRP5 and subsequently with either pNP4 or pREN88 using electroporation at 1.8 kV with a time constant value of 3.5–4 ms. After a recovery in LB media without antibiotics the transformants were plated on plates supplemented with 35 μg/ml chloramphenicol and 100 μg/ml ampicillin.

TolQ-mCherry (C-terminal fusion) expressing strains PB168 and PB177 (with and without a kanamycin cassette, respectively)^19^, were transformed with pRP5 for microscopy experiments on TolQ diffusion and clustering.

*E*. *coli* DPD1718 is a *lux* reporter strain used previously to monitor colicin-mediated DNA damage in vivo^36^, where the *E*. *coli recA* promoter region is fused to the *Photorhabdus luminescens luxCDABE* operon integrated into the *lacZ* locus of *E*. *coli* DPD1692^37^.

### Colicin purification and fluorophore labelling

The construction, expression and purification of Y324C, L447C, K469C ColE9 (which contains an internal disulphide bond that blocks cell entry) and Δ1-83 Y324C, L447C, K469C ColE9 have been described elsewhere^16^. Cys469 in the C-terminal DNase domain of these ColE9 constructs was labelled with a 20-fold molar excess of fluorophore (Alexa Fluor 594-maleimide (Invitrogen) or TMR-maleimide (Sigma)) as previously described^16^. The labelling efficiency (typically 0.8 fluorophores/protein) was estimated spectrophotometrically (Alexa Fluor 594, *ε*_max_ = 90,000 cm^−1^ M^−1^; TMR, *ε*_max_ = 80,000 cm^−1^ M^−1^) and colicin (ColE9, *ε*_280 nm_ = 46,075 cm^−1^ M^−1^) concentrations after correcting for absorption at 280 nm by the fluorophore (Alexa Fluor 594, *A*_280 __nm_ = 0.56 × *A*_590 nm_; TMR, *A*_280 nm_ = 0.3 × *A*_547 nm_). Analysis of single-molecule photobleaching characteristics for fluorophore-labelled colicins adsorbed on a microscope slide surface and viewed by TIRFM were consistent with labelling at a single position.

### Plate and cell growth assays

For plate assays, 5 µl of cells from exponential phase in LB broth (supplemented with 1 mM arabinose and antibiotics when required) were spotted on LB-agar plates with or without 2% (w/v) SDS (also supplemented with 1 mM arabinose and antibiotics where required), using five serial dilutions (from 10^−2^ to 10^−6^). Plates were incubated overnight at 37 °C before imaging on a Syngene GBox gel documentation system.

*E*. *coli* cells were grown at 37 °C in LB broth (supplemented with 1 mM arabinose and antibiotics where required). When optical density at 600 nm reached 0.6, ColE9 (300 nM) in combination with Polymyxin B (144 nM; Sigma-Aldrich) or EDTA (1.5 mM; Sigma-Aldrich) were added to the media. Optical density was then monitored every 30 min for 4 h. In parallel, samples from treated and untreated cells were transferred in microscopy chambers between an agar pad and a coverslip. DIC images of individual cells were taken every 30 min for 1.5 h to check for growth and division defects. Cell length was defined as the distance from one pole of a bacterium to the other and measured as a straight line on at least 30 cells per condition using ImageJ software. All experiments were done in triplicate. All curves and scatter plots were constructed using GraphPad software (Prism V).

SOS stress response assays consisted of growing *E*. *coli* DPD1718/pNP4, pRP5 cells in 96 well plates to OD (600 nm) 0.4 at 37 °C with orbital shaking, using a 1 in 1000 dilution from an overnight culture and then adding LB or ColE9 (300 nM) or mitomycin (2 mM). OD (600 nm) and bioluminescence (490 nm) was then monitored in triplicate, for 3 h, every 10 min, using a TECAN Genios Pro 96/384 Multifunction Microplate Reader.

### Preparation of microscopy samples for live cell imaging

Cells were grown at 37 °C in LB media to exponential phase, after which 200 ml of cells were transferred to 4 ml supplemented M9-glucose media (0.1 mM CaCl_2_, 0.1 mM FeSO_4_, 2 mM MgSO_4_, 1 g/l NH_4_Cl, 0.05% (w/v) casamino acids, 0.4% (w/v) d-glucose). Strains transformed with pNP4 plasmid expressed leaky GFP-TolA. For the transient expression of BtuB, cultures were grown in the presence of 1 mM arabinose for 2 h. Cells were brought to an OD_600_ of 0.15 in 200 μl, centrifuged at 4711×*g* for 3 min to pellet cells, and resuspended in 200 μl supplemented M9-glucose. Cells were treated with 300 nM labelled ColE9 or Δ1-83ColE9, both containing an internal disulphide bond to block cell entry, for 10 min at room temperature in a foiled micro-centrifuge tube. After colicin treatment, cells were pelleted at 4711×*g* for 3 min and resuspended in 200 μl supplemented M9-glucose. This washing step was repeated twice to remove excess fluorescent protein. Immediately before or after colicin treatment, cells were treated with 0.1 mM CCCP for 20 min to decouple the pmf. Trypsin treatment consisted of adding 35 μg/ml of protease to the sample for 20 min prior to mounting cells between an agar pad and a coverslip. The agar pad was made with 200 µl of M9-glucose containing 1% UltraPure™ agarose (w/v), introduced into a 1.5 cm × 1.6 cm Gene Frame matrix (Thermo Fischer Scientific) that was previously adhered to a clean slide. When disulfided ColE9 was incorporated to the agar pad, colicin was introduced at 3 µM final concentration in M9-glucose containing 1% UltraPure™ agarose (w/v) during the cooling step of the gel. The agar pad was formed by addition of a clean coverslip on top until solidification had occurred. 10 µl of stained bacteria were then added to the pad, which was sealed using a clean coverslip.

### Immunostaining

Cells were grown in M9-glucose media as described above and fixed in 4% (v/v) formaldehyde in PBS for up to 20 min at 4 °C followed by 10 min permeabilization in 0.1% (v/v) Triton X-100 in PBS at room temperature. Permeabilized bacteria were then washed in PBS and incubated for 30 min at room temperature with rabbit polyclonal anti-TolA antibody (provided by Daniel Walker, University of Glasgow) at 50 µg/ml final concentration in PBS supplemented with 1% (w/v) BSA. After a further wash step in PBS, bacteria were incubated for 30 min at room temperature with donkey anti-rabbit antibody^AF555^ (Invitrogen) at 25 µg/ml final concentration in PBS. Cells were then washed twice in PBS and mounted between an agar pad and a coverslip for confocal microscopy acquisition.

### FRAP acquisition

Measurements were taken using a Zeiss LSM 780/Axio Examiner Z1 motorised upright laser scanning microscope equipped with Argon multiline 458/488/514 nm (25 mW), and HeNe 561 nm (1 mW). Optical magnification was provided by a ×100 oil-immersion objective (Zeiss, NA 1.4). Images were recorded by scanning the laser over a 13.5 × 13.5 μm area with the image size set to 512 × 512 pixels, scan speed set to 7 (3.15 μs per pixel) and a digital zoom of ×10. Images were recorded using Zeiss Zen 2011 software.

Bleaching of fluorescently labelled proteins was performed at 37 °C using 20 scan iterations over a rectangular ROI (region of interest, 50 × 30 pixels) and the corresponding laser power set to 100%. To limit photobleaching thereafter, FRAP was monitored at 1–3% of maximum laser power. The diameter of the pinhole was varied between 0.2–0.3 μm depending on the sample conditions. One image was recorded prior to bleaching GFP or mCherry fluorescence. Fluorescence images were acquired at 5, 10, 15, 20, 25, 30, 35, 40, 45, 50, 55, 60, 120, and 180 s post-bleach using 10× digital gain. A differential interference contrast (DIC) microscopy image was acquired before and after each FRAP experiment to ensure the image of the bacterial cell remained in focus. In all cases, DIC images were recorded at 0.1% of maximum power. Images were analysed using ImageJ software (version 1.48; Wayne Rasband, National Institutes of Health, Bethesda, MD). TIFF stacks were created from lsm files obtained from Zeiss Zen 2011 and if required, aligned either manually or by using the stackreg plugin for ImageJ if a second cell was present.

### TIRFM acquisition

Live cell single-molecule tracking was performed at room temperature on a custom-built TIRF microscope built around the Rapid Automated Modular Microscope (RAMM) System (ASI Imaging). Laser lines of 488 and 561 nm provided by a multi-laser engine were used to activate GFP and TMR/mCherry, respectively (iChrome MLE, Toptica). Laser beams were collimated at the fibre output and focused (×100 oil-immersion objective, NA 1.4, Olympus) onto the sample at an angle allowing for highly inclined thin illumination^38,39^. Fluorescence emission was filtered by a dichroic mirror and notch filter (ZT405/488/561rpc & ZET405/488/561NF, Chroma) and then projected onto an EMCCD camera (iXon Ultra, 512 × 512 pixels, Andor). The pixel size was 96 nm in the magnified image. Transmission illumination was provided by an LED source and condenser (ASI Imaging). Sample position and focus were controlled with a motorised piezo stage, a *z*-motor objective mount, and autofocus system (MS-2000, PZ-2000FT, CRISP, ASI Imaging). All video data were collected at room temperature (20–22 °C), with a 33 ms frame rate. Moderate bleaching of the sample was applied to be able to track single molecules, as described below.

### SIM acquisition

Live cells were imaged at room temperature with a ×60, NA 1.42 oil objective on a Deltavision OMX V3 Blaze (GE) equipped with 488 and 592 nm lasers. Spherical aberration was minimised by choosing an immersion oil with a refractive index giving symmetrical point spread functions and image stacks of several μm thickness taken with 0.125 μm *z*-steps and 15 images (three angles and five phases per angle) per *z*-section and a 3D-structured illumination with stripe separation of 213 nm and 238 nm at 488 nm and 594 nm, respectively. Laser power of 10% was used for both laser lines and the sample scanned over 4 µm thickness (32 slices). Image stacks were reconstructed using Deltavision softWoRx 6.1.1 software with a Wiener filter of 0.002 using wavelength specific experimentally determined OTF functions^40^. This leads to a halving of the pixel size and a doubling of the *X* and *Y* direction pixel number (ie, four times as many pixels), but no change in *z*-steps in the image stacks. For two-colour experiments, reference *z*-stacks of 200 nm TetraSpeck beads (Life Technologies, T7280) were aligned with custom rigid body alignment routines correcting for *x*, *y* and *z* shifts, magnification and rotation differences between channels. The transformations were then applied to align the two-channel images.

### Image analysis

FRAP was quantified using the ‘FRAP norm’ plugin for ImageJ. For this analysis, mean fluorescence intensities of the ROI, background, reference region (unbleached part of cell) and the whole cell were used. All measurements except for that of the whole cell were performed using a 50 × 50 pixel box. Gradual photobleaching that occurred during image acquisition was compensated for by normalising the ROI fluorescence to the mean fluorescence of the entire cell in the same image. In addition, the mean intensity of the ROI in the post-bleached images was normalised to the mean ROI pre-bleach intensity. Background fluorescence (mean fluorescence outside of cells) was also used for ‘double normalisation’^41^. More than 30 cells were analysed in this way per condition, in triplicate. All TIRFM videos were analysed using custom software (PATRACK), implemented in visual C++ and provided by Milhiet and Dosset^42^. The centre of each fluorescence peak was determined with sub-pixel resolution by fitting a two-dimensional elliptical Gaussian function. The two-dimensional trajectories of single molecules were constructed frame-by-frame, selecting particles that displayed a single bleaching step. Diffusion coefficient values were determined from a linear fit to the mean square displacement (MSD) plots between the first and fourth points according to the equation: MSD(*t*) = 4Dt. These experiments and analyses were done in triplicate, with at least 30 trajectories per condition.

From SIM images, intensity quantification was obtained using ImageJ. Within each cell, the maximum and the mean intensity were determined after normalising with background fluorescence. Relative intensity corresponds to the ratio of the maximum intensity and the mean intensity within a cell, which would equal 1 in a perfect homogenous pattern and would increase with fluorescence heterogeneity (ie, clustering). More than 30 cells were analysed this way per condition, in triplicate. Co-clustering analysis calculates the percentage of TolA clusters that overlap significantly with ColE9/OMP clusters in each experimental condition. Significance in this instance is taken to mean where the OMP and IMP fluorophores share at least 3 pixels. By contrast, >95% of free (ie, non-coupled) fluorophores at a similar density would share <3 pixels if distributed randomly.

CoLocalizer Pro 2.7.1 software (CoLocalization Research Software, http://www.colocalizer.com) was used for co-localisation of red and green channels for TIRFM. The co-localisation value corresponded to the ratio between yellow pixels over the sum of yellow, red and green pixels on each overlapped picture. TIRFM images resulted from 100 consecutive frame stacks in both channels and 2D SIM images resulted from the slice displaying maximum cell length (defined as the middle of the bacterial *Z* plane). Experiments were done in triplicate, with at least 30 cells per condition.

Distribution of fluorescence intensity along the *x*-axis of bacteria was determined using a custom script implemented in MATLAB (version 2012a, MathWorks)^16^. Raw images were initially thresholded against a user-defined intensity (selection via a graphical user interface) to provide a binary image, approximately highlighting bacteria against the background. High frequency noise was reduced through application of the built-in MATLAB medfilt2 median filter. Bacterial poles were identified as corresponding to the 2 pixels in a continuous region with the largest separation. Intensity along the selected bacteria was measured 11 times between the bacterial end-points, each at a uniformly spaced offset from the bacterial long axis. The mean profile was calculated and normalised to the range 0–100 for comparison between bacteria. A total of 30 cells were analysed this way per condition, in triplicate. All data were plotted in GraphPad software (Prism V).

### Data availability

The data supporting the findings of the study are available in this article and its Supplementary Information files, or from the corresponding authors upon request.

## Electronic supplementary material

Supplementary Information

Description of Additional Supplementary Files

Supplementary Movie 1

Supplementary Movie 2

Supplementary Movie 3

Supplementary Movie 4
