# Supplementary material for: Intermembrane crosstalk drives inner-membrane protein organization in Escherichia coli
Source: Nat Commun. 2018 Mar 14;9:1082. doi: 10.1038/s41467-018-03521-4 (PMC5852019; doi:10.1038/s41467-018-03521-4)
Supplement: Supplementary file 1 — Supplementary Information [file 41467_2018_3521_MOESM1_ESM.pdf]

Supplementary Information PDF

Intermembrane crosstalk drives inner membrane protein organization in *Escherichia coli*

Rassam et al

Supplementary Information

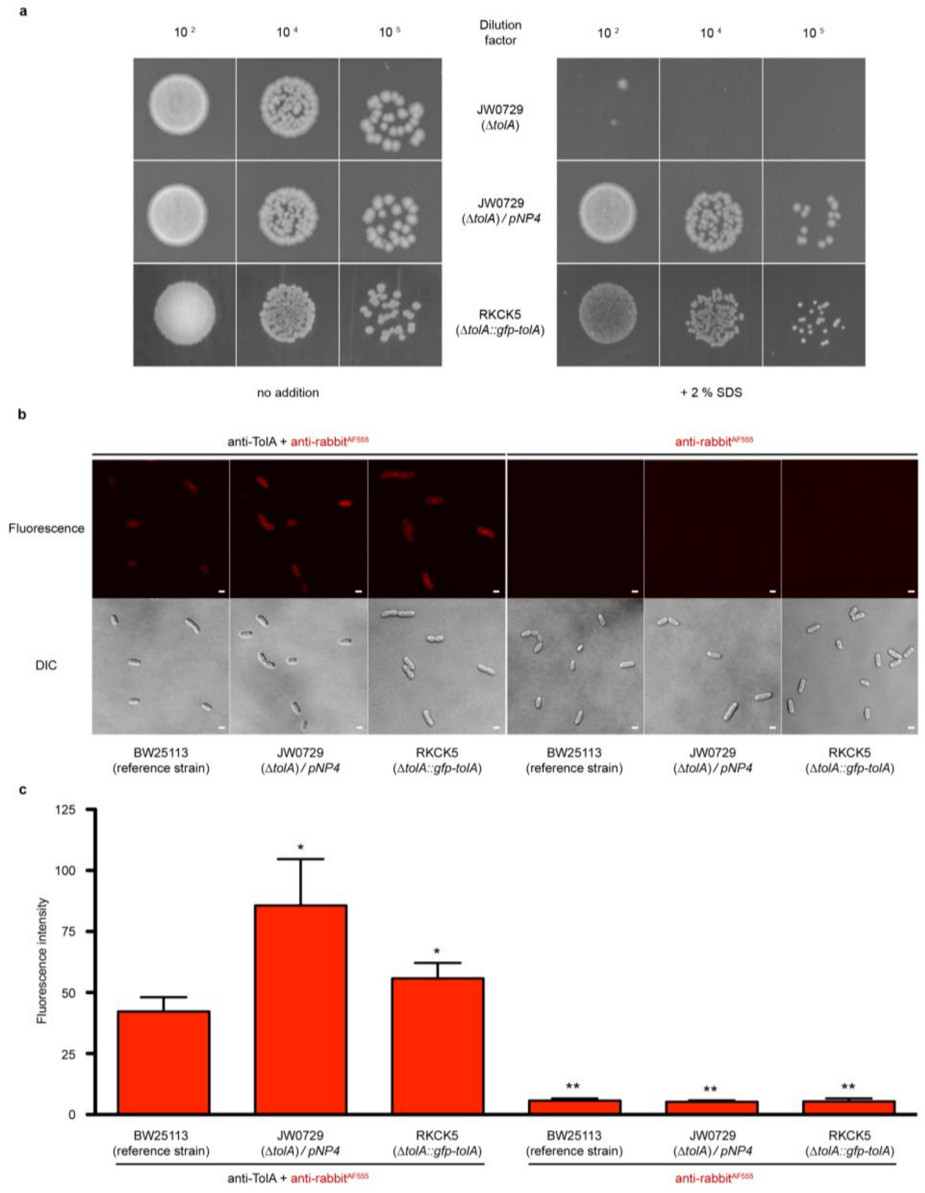

Supplementary Figure 1. Complementation of *E. coli* Δ*tolA* cells by plasmid or chromosomal copies of *gfp-tolA*. (**a**) *E. coli* JW0729, a Δ*tolA* strain, was complemented with *gfp-tolA* expressed from a non-induced plasmid (pNP4)[14] and compared to *E. coli* RKCK5 in which was inserted a chromosomal copy of *gfp-tolA* (see Methods for details). Strains were grown in the presence or absence of 2% (w/v) SDS on LB agar plates. JW0729 cells have a destabilized outer membrane and so are unable to grow in the presence of SDS. *gfp-tolA* expression restores OM

stability and growth on SDS. (**b**) Confocal microscopy images of BW25113 (*first and fourth columns*), JW0729 complemented with pNP4 (*second and fifth columns*) and JW0729 complemented with a chromosomal copy of *gfp-tolA* (*third and sixth columns*). Cells were fixed with 4 % PFA (v/v), permeabilized with 0.1 % Triton X100 (v/v) and labelled with secondary anti-rabbit^{AF555} following the addition of anti-TolA antibody (*columns one to three)*. Controls (*columns four to six)* did not include addition of anti-TolA antibody. (**c**) Quantitation from immunofluorescence images in panel *B* of GFP-TolA levels relative to wild type TolA in BW25113 cells and JW0729 complemented with a chromosomal copy of *gfp-tolA* (RKCK5 cells). Leaky expression from pNP4 results in 2-3-fold elevation in the levels of GFP-TolA relative to wild-type TolA whereas the chromosomal copy gave 1-2-fold increase compared to the reference strain. Controls where anti-TolA antibody was not used showed background levels of immunofluorescence. Error bars correspond to SD for fluorescence intensity from 45 cells. Asterisk in histograms, p value <0.01 from a student t test.

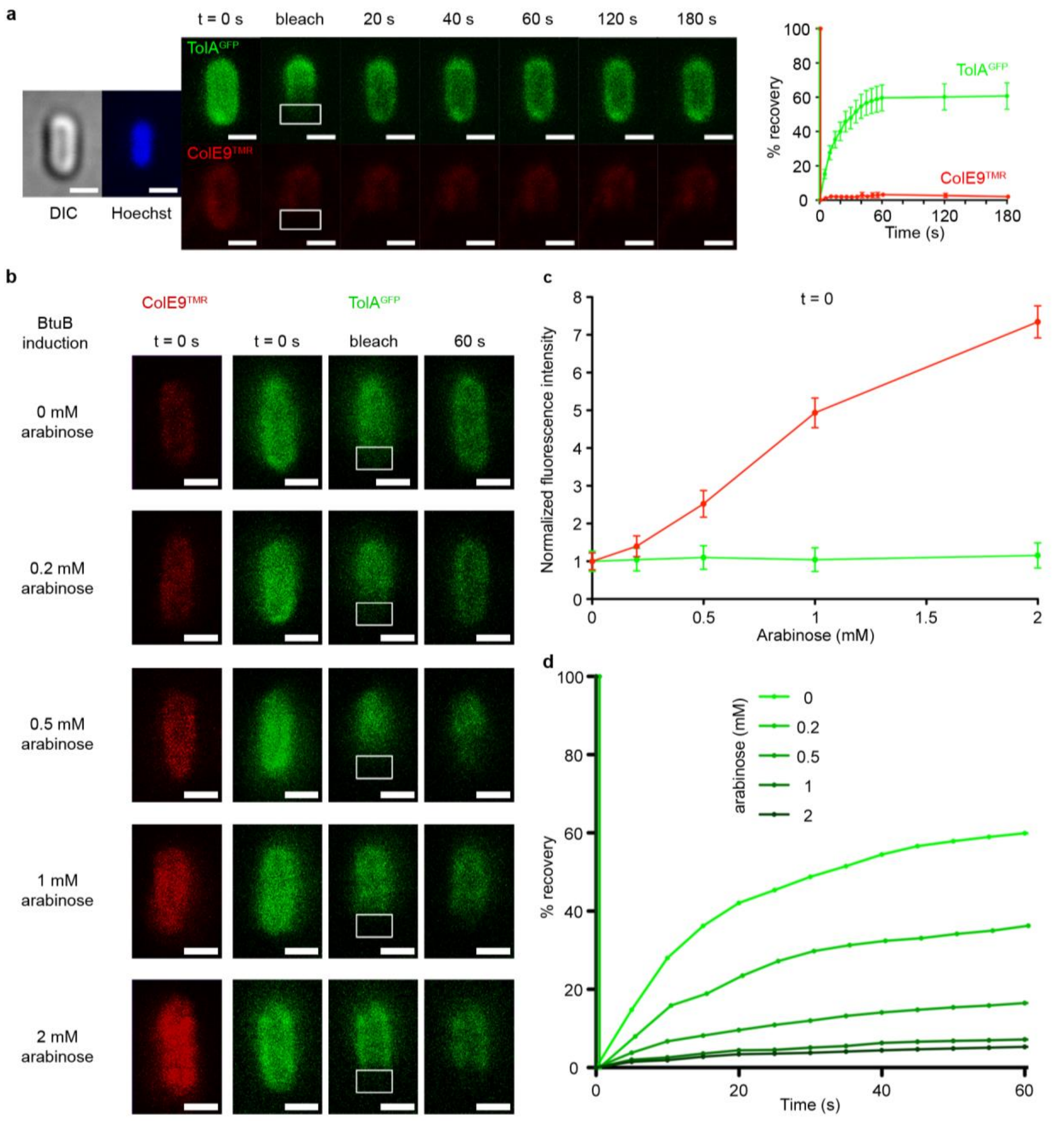

Supplementary Figure 2. Levels of BtuB in the OM determine restriction imposed on GFP-TolA mobility in the IM by ColE9^{TMR}. (**a**) FRAP data for ColE9^{TMR} bound to BtuB in the OM and GFP-TolA in the IM in JW0729 cells transformed with pNP4 (GFP-TolA) and pRP5 (BtuB) plasmids, without arabinose induction. Sequential FRAP images over time (in seconds) are displayed (*centre panels*), along with the corresponding DIC and Hoechst-stained chromosomal DNA images (*left*), and recovery curves (*right*). (**b**) JW0729/pNP4, pRP5 cells were

incubated with increasing concentrations of arabinose to induce BtuB production. ColE9^{TMR} (300 nM) was added to cells and then red (ColE9^{TMR}) and green (GFP-TolA) fluorescence visualized on agar pads. Panels show time-points from FRAP experiments of GFP-TolA fluorescence. At low concentrations of arabinose fluorescence recovers after photobleaching but fails to recover as the amount of BtuB in the OM increases. Scale bars, 1 µm. (**c**) Quantification of ColE9^{TMR} binding to JW0729/pNP4, pRP5 cells with increasing arabinose concentration (*red line*). Also shown are the normalized fluorescence levels for GFP-TolA (*green line*) in the same cells. Error bars correspond to SD from 3 experiments (n = 15). (**d**) GFP-TolA fluorescence recovery curves showing how ColE9^{TMR} addition to cells increasingly restricts the mobility of GFP-TolA in the IM.

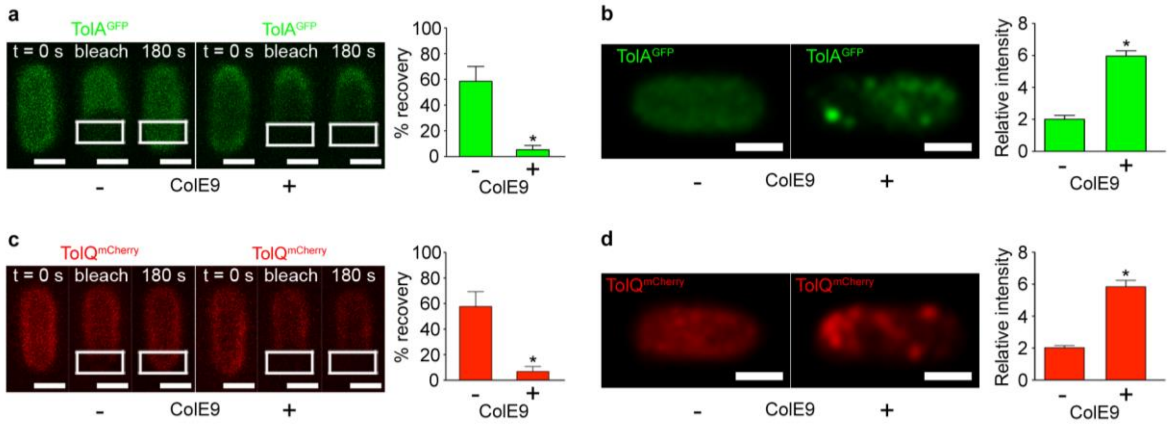

Supplementary Figure 3. ColE9 restricts the mobility and forms IM clusters of TolQ-mCherry and GFP-TolA. Data shown for gene fusions expressed from the chromosome. (**a, c**) Confocal FRAP data and (**b, d**) TIRFM experiments showing slow diffusion and clustering into small islands of (**a, b**) GFP-TolA in RKCK5/pRP5 cells and (**c, d**) TolQ-mCherry in PB168/pRP5 cells in the presence of ColE9 (where indicated) and BtuB induction. Both histograms shown were averaged from 15 bacteria. Error bars correspond to SD from 3 experiments. Scale bars, 1 µm. Asterisk in histograms, p value <0.01 from a student t test.

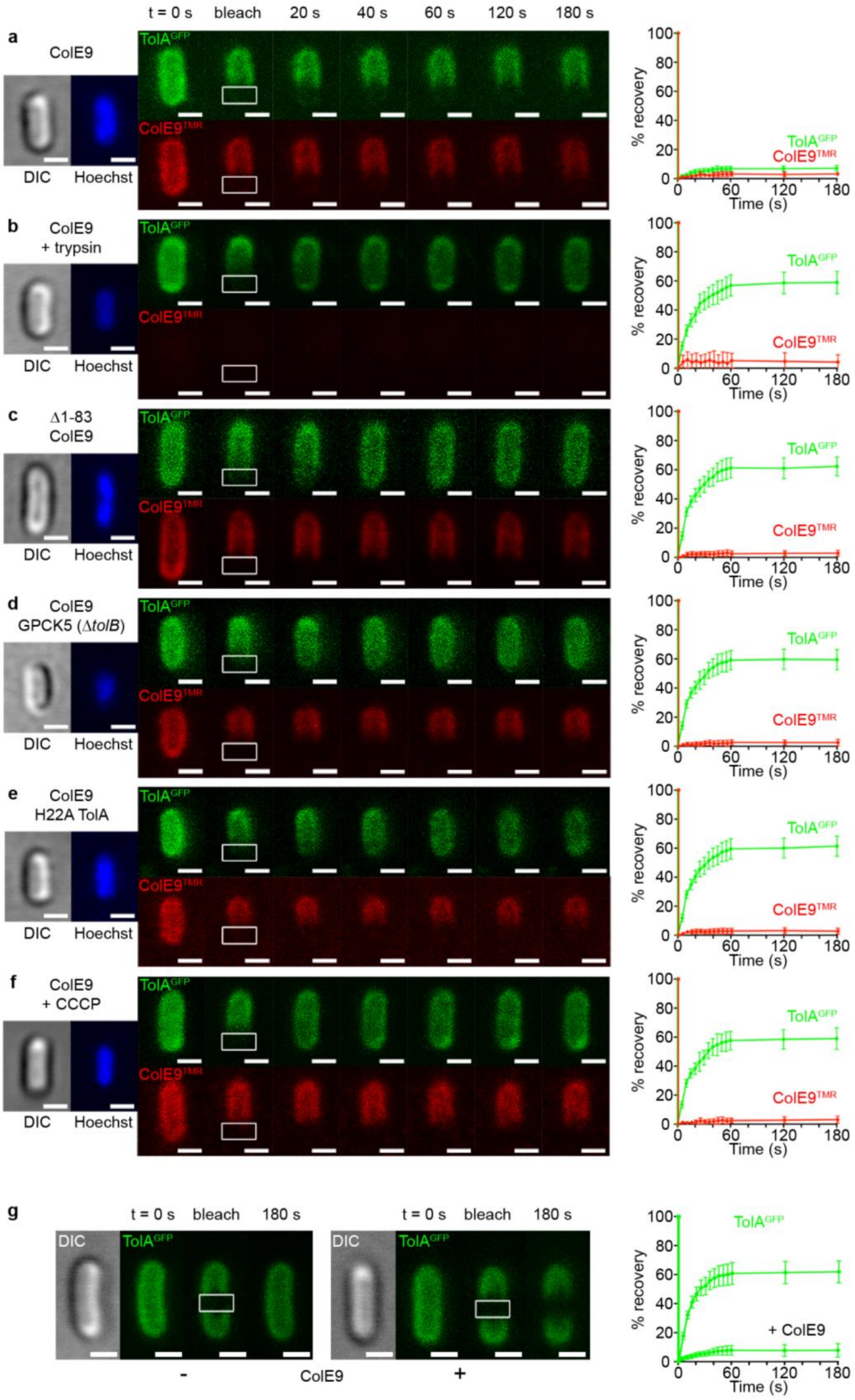

Supplementary Figure 4. FRAP data establishing the requirement for a transenvelope bridge and the PMF for ColE9^{TMR}/OMP-mediated capture of GFP-

TolA in the IM. Panels a-f show FRAP data for ColE9^{TMR} bound to BtuB/OmpF in the OM of *E. coli* JW0729/pNP4, pRP5 cells expressing GFP-TolA (not induced) and BtuB (induced), respectively. Sequential FRAP images over time (in seconds) are displayed (*centre panels*), along with the corresponding DIC and Hoechst-stained chromosomal DNA images (*left*), and recovery curves (*right*). (**a**) ColE9^{TMR} added to cells expressing GFP-TolA. No recovery of red or green fluorescence is seen after 180 s indicating immobility of both the outer and inner membrane proteins. (**b**) Trypsin degrades surface-bound ColE9^{TMR}, which releases TolA leading to the recovery of fluorescence for GFP-TolA in the IM and loss of all ColE9^{TMR} fluorescence at the OM. (**c**) Δ1-83 ColE9^{TMR} added to GFP-TolA cells. Δ1-83 ColE9^{TMR} cannot bind TolB in the periplasm (main text, Figure 1A), which leads to the recovery of GFP-TolA fluorescence due to increased mobility in the IM. (**d**) Addition of ColE9^{TMR} to GPCK5/pNP4, pRP5 cells results in recovery of GFP-TolA fluorescence. TolB is required to mediate the interaction of ColE9 with TolA in the IM (see Figure 1A). (**e**) Addition of ColE9^{TMR} to JW0729/pREN88, pRP5 cells expressing GFP-H22A TolA (uninduced) and BtuB (induced), respectively. His22 is located in the transmembrane helix of TolA and is involved in shuttling protons across the membrane in association with TolQ and TolR (Figure 1a). (**f**) Addition of the uncoupler CCCP (0.1 mM), which abolishes the PMF, blocks the ability of ColE9^{TMR} to restrict the mobility of GFP-TolA in the IM. (**g**) FRAP experiments with (+) and without (-) ColE9 demonstrating that the change in fluorescence recovery also occurs within the central regions of cells when ColE9 forms its translocon complex. Error bars correspond to SD from 3 experiments, n = 15. Scale bars, 1 µm.

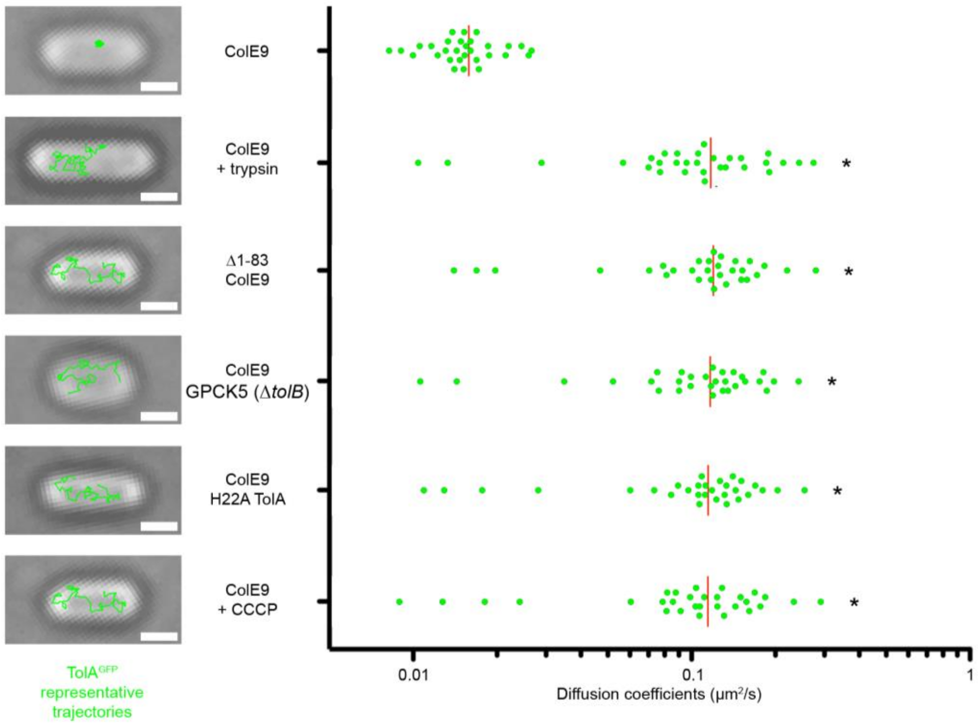

Supplementary Figure 5. Single particle tracking (SPT-) TIRFM data showing how restricted lateral mobility of GFP-TolA is abolished when the connection between the OM and IM is broken and coupling to the PMF is disrupted. Representative GFP-TolA trajectories (*left*) from SPT-TIRFM experiments on JW0729/pNP4, pRP5 cells (panels 1, 2, 3 and 6), GPCK5/pNP4, pRP5 cells (panel 4) and JW0729/pREN88, pRP5 cells (panel 5) with leaky expression of GFP-TolA or its mutant and induced BtuB. Scatter plot of corresponding diffusion coefficients showing confinement of GFP-TolA when unlabelled ColE9 is added and unrestricted diffusion for all other treatments as shown in Supplementary Figure 4. Quantification of the MSD(t) for every GFP-TolA molecule tracked yielded a lateral diffusion coefficient which is shown in the scatter plot (n = 30 cells) for each condition. Scale bars, 1 µm. Asterisks, p value <0.001 from a Mann-Whitney U test.

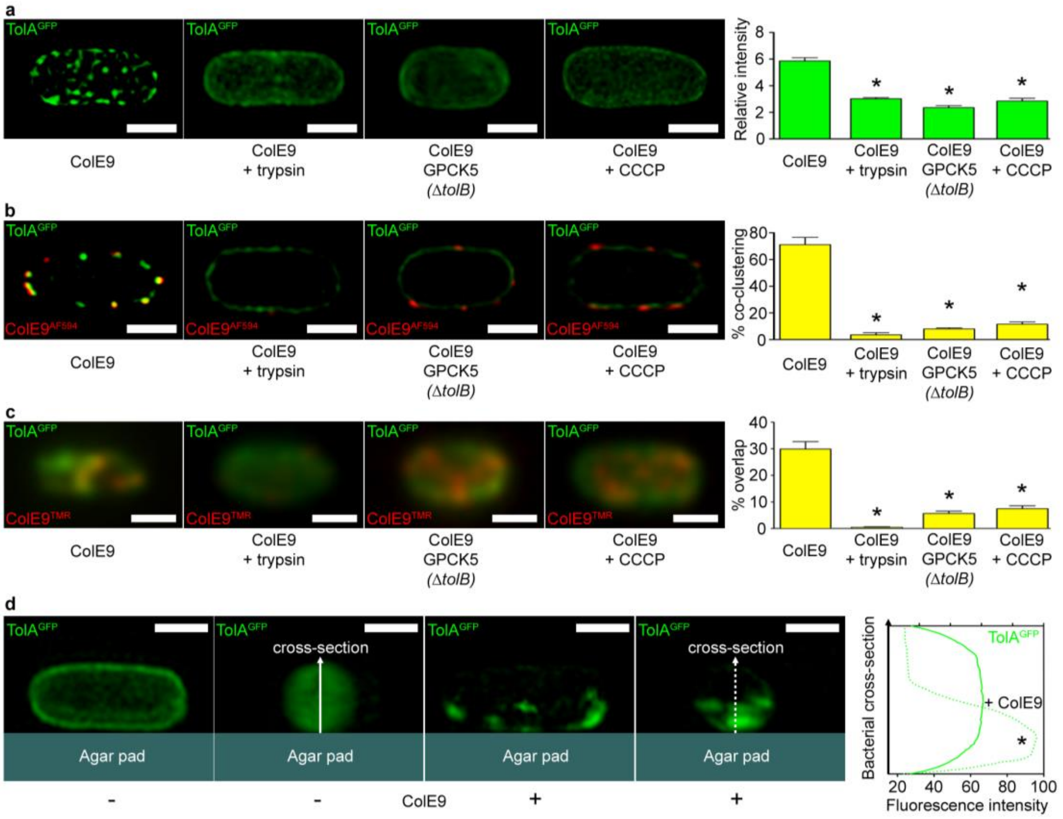

Supplementary Figure 6. Dissecting ColE9/OMP-induced clustering of TolA visualized by 3D-SIM, 2D-SIM and TIRFM. (a) 3D-SIM images and associated statistics showing that GFP-TolA fluorescence intensity becomes homogeneous when trypsin is used to proteolyse surface-bound ColE9 (for *E. coli* JW0729/pNP4, pRP5 cells expressing GFP-TolA (not induced) and BtuB (induced), respectively), when *tolB* is absent in *E. coli* GPCK5/pNP4, pRP5 cells, or when the PMF is abolished through the addition of CCCP. In contrast to experiments shown in Figure 3 (main text), CCCP was added after the formation of ColE9-induced clusters hence the PMF is required both to initiate confinement of GFP-TolA and to maintain it. (**b**) 2D-SIM data with associated statistics for cells/treatments as for panel a, showing how co-localization of GFP-TolA in the IM and ColE9^{TMR} in the OM within islands (*yellow fluorescence*) requires intact ColE9, TolB and PMF. (**c**) TIRFM data with associated statistics for cells/treatments as for a showing how co-localization of GFP-

TolA in the IM and ColE9^{TMR} in the OM within islands (*yellow fluorescence*) requires intact ColE9, TolB and the PMF. (**d**) 3D-SIM images (*left*) of JW0729/pNP4, pRP5 cells expressing GFP-TolA after immobilisation on agar pads impregnated with (+) ColE9 (3 µM) or in its absence (**-**). ColE9-induced GFP-TolA clustering occurs only on the side of the cell in close proximity to the ColE9-impregnated agar pad. Average fluorescence intensity data for vertical cross-sections of bacteria are also shown; with (*continuous line*) or without (*dashed line*) ColE9 incorporated in the agar pad. Asterisk in histograms, p value <0.01 from a student t test (n = 15 cells). Asterisk in curve plot, p value <0.001 from a Mann-Whitney U test. Scale bars, 1 µm.

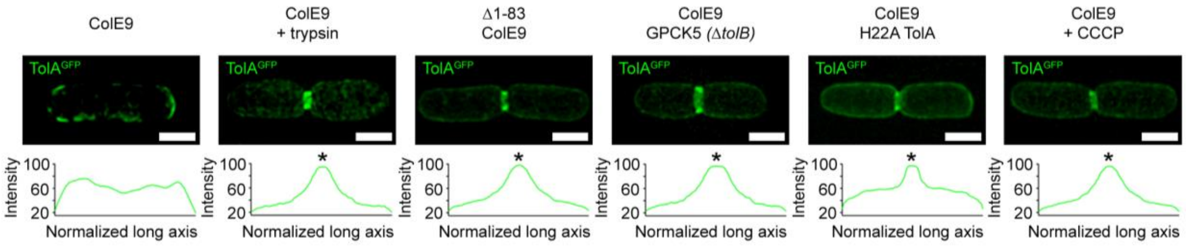

Supplementary Figure 7. ColE9/OMP-mediated translocon formation impedes recruitment of TolA to the septum during cell division. 3D-SIM data of JW0729/pNP4, pRP5 cells (*columns 1, 2, 3 and 6*), GPCK5/pNP4, pRP5 cells (*column 4*) and JW0729/pREN88, pRP5 cells (*column 5*). In all cases, only uninduced GFP-TolA or GFP-H22A TolA fluorescence was observed in cells where BtuB was induced. Images where collected after 20 min of growth in the presence of ColE9^{TMR} and the effect of various treatments/mutations. Disruption of the transenvelope complex linking ColE9 bound at the OM to BtuB/OmpF with GFP-TolA in the IM (trypsin, Δ1-83 ColE9, Δ*tolB*, H22A TolA, CCCP) releases TolA so it can be recruited to the septum. Average intensity data (n = 9 cells) for each condition is shown beneath each cell. Asterisk, p<0.001 from a Mann-Witney U test. Scale bars, 1 µm.

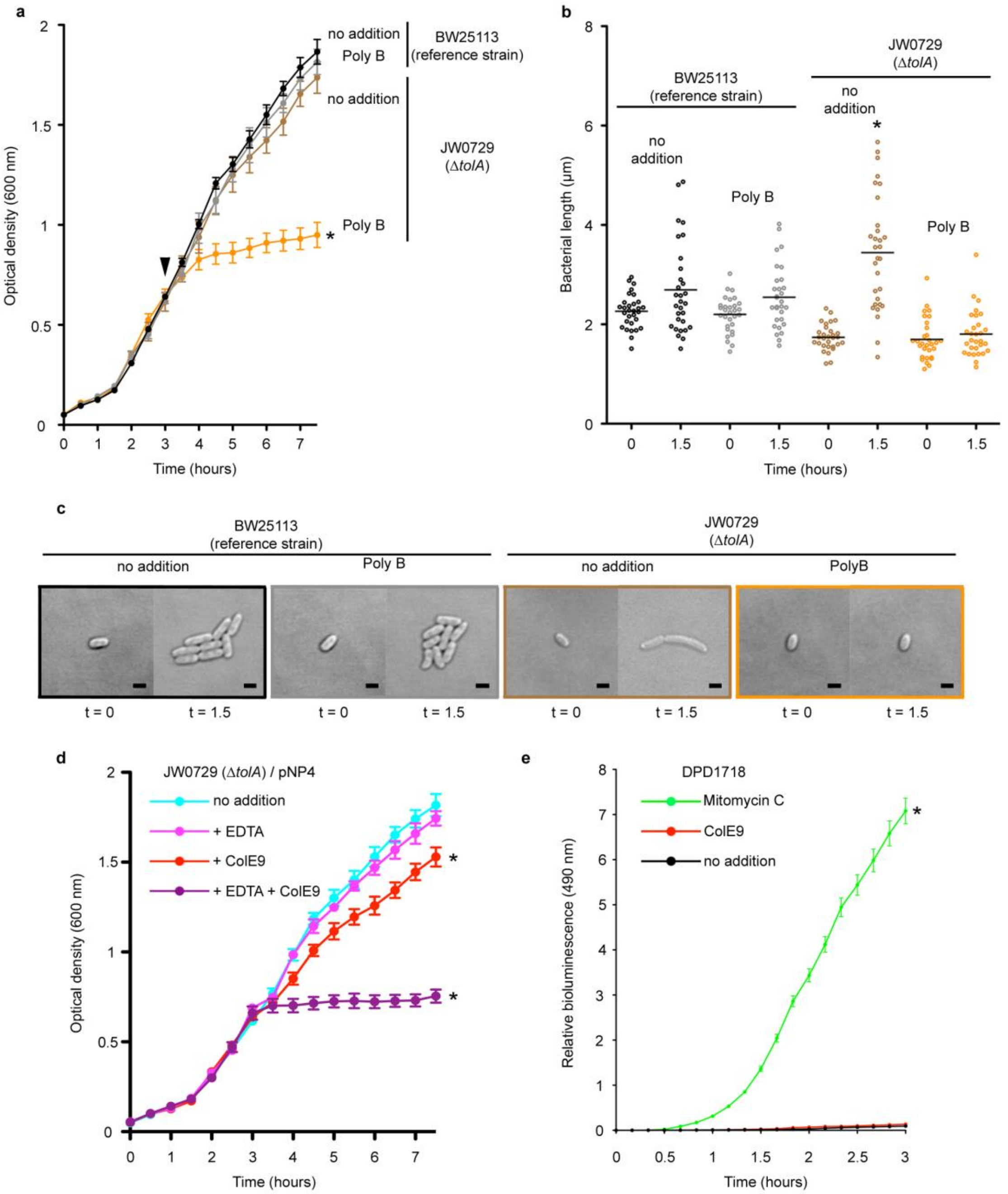

Supplementary Figure 8. ColE9/OMP-mediated capture of GFP-TolA mirrors the effects of a *tolA* deletion phenotype but does not activate the SOS response. (**a**) Growth curves for *E. coli* BW25113 (reference strain) and JW0729 (Δ*tolA*) in the presence and absence of polymyxin B (0.14 µm). *Color code*: BW25113 without treatment (*black*); BW25113 plus polymyxin B (*grey*); JW0729 without treatment (*brown*); JW0729 with polymyxin B (*orange*). Arrow indicates point of polymyxin B addition. Asterisk, p value <0.01 from a student t test from 3

experiments. (**b**) Bacterial length data for cells treated as in panel a (same color coding). Two sets of cell length data are shown for each treatment, 0 h and after 1.5 h growth on agar pads at 37ºC. Asterisk, p value <0.001 from a Mann-Whitney U test for 30 cells. (**c**) Representative DIC images of BW25113 and JW0729 Δ*tolA* cells in the presence and absence of polymyxin B, as described in panel b, after 0 and 1.5 h growth on an agar pad. *E. coli* JW0729 cells show characteristic filamenting phenotypes that are also seen when GFP-TolA is sequestered by OMP-bound ColE9 (Figure 4, main text). (**d**) Growth curves for JW0729/pNP4, pRP5 cells expressing GFP-TolA (not induced) and BtuB (induced), respectively, showing that the ColE9 transenvelope complex also induces OM instability toward the chelating agent EDTA (1.5 mM). (**e**) The ColE9 translocon does not activate the SOS stress response in *E. coli* cells. Panel shows bioluminescence data for *E. coli* DPD1718/pRP5 cells where lux luminescence is linked to the SOS response (see Methods). Addition of disulfided ColE9 (300 nM) does not induce lux luminescence in contrast to the DNA damaging agent mitomycin C (2 mM).

Supplementary Table 1. PCR primers used in this study

Application	Primer sequence (5' to 3')
TolA H22A mutagenesis in pNP4	TCAGCAGTGCTGGCTGTCATCTTATTTGCGGC<br>GCTGATCTG (FWD)<br>CAGATCAGCGCCGCAAATAAGATGACAGCCAG<br>CACTGCTGA (REV)
Amplification of *cat* (Cam^r) from pACYC184	AGAAGCCACTGGAGCACCTC (FWD)<br>GGAGAGCCTGAGCAAACTGG (REV)
Excision of *bla* (Amp^r) from pNGH15	GAGGTGCTCCAGTGGCTTCTCAAAAGAGTTTG<br>TAGAAACGC (FWD)<br>TGTCAGACCAAGTTTACTCATCCAGTTTGCTCA<br>GGCTCTCC (REV)
Amplification of 300 bp upstream *tolA* from BW25113	ATCGATGAATTCTTGTTGAAGTGTCTGGTATTG<br>GTCAGTACACCGTGGTGGT (FWD)<br>GCTCCAGCACCGGTTCTCGGTTTCCAAAAACT<br>GTTCGCCTGTTACCCGCTCTCT (REV)
Amplification of P_B with overhang to 300 bp downstream *tolB* from BW25113	CCATTGGACTTCAAAGAGCTCTCGCGATGTTG<br>ACTG (FWD)<br>TTCAATTAATTATTAATCTCCCTTATCTGGACCG<br>A (REV)
Amplification of 300 bp downstream *tolB* with overhang to P_B from BW25113	TAATAATTAATTGAATAGTAAAGGAATCAT<br>(FWD)<br>ATCCCTACTAGTTCCAGCATTTGAGCGAAGTCA<br>GAACGGATA (REV)
Abolishment of MunI restriction within *gfp* in pNP4	CCATTATCAACAAAATACTCCTATTGGCGATGG<br>CCCTGTCC (FWD)<br>GGACAGGGCCATCGCCAATAGGAGTATTTTGT<br>TGATAATGG (REV)
Amplification of *gfp-tolA* from pNP4	GCTCCAGCACCGGTATGAGTAAAGGAGAAGAA<br>CTTTTCACTGGAG (FWD)<br>ATCGATCAATTGTTACGGTTTGAAGTCCAATGG<br>CGCG (REV)
Deletion of AgeI restriction site in pREN98	CAGTTTTTGGAAACCGAGAATGAGTAAAGGAG<br>AAGAAC (FWD)<br>GTTCTTCTCCTTTACTCATTCTCGGTTTCCAAAA<br>ACTG (REV)
Amplification of 300 bp downstream *tolA* from BW25113	GCGCGCGAGCTCTCGCGATGTTGACTGTTCGG<br>ACGGTCAACATCA (FWD)<br>ATCCCTACTAGTGCCGCCAATATCTTCAGGTG<br>CCGCACCAGGCCC (REV)

FWD: forward primer, REV: reverse primer

Supplementary Table 2. *E. coli* strains used in this study

Strain name	Genotype	Reference
BW25113	F-, Δ(*araD*-*araB*), Δ*lac*(::*rrnB*-3), *λ*-, *rph*-1, Δ(*rhaD*-*rhaB*), *hsdR*514	(35)
JW0729	F-, *Δ(araD-araB)*, *ΔlacZ*(::rrnB-3), *ΔtolA::kan*, λ^-, *rph-1*, *Δ(rhaD-rhaB)*, *hsdR514*	(33)
GPCK5	F-, *Δ(araD-araB)*, *ΔlacZ*(::rrnB-3), Δ*tolAtolB*::*kan*, λ^-, *rph-1*, *Δ(rhaD-rhaB)*, *hsdR514*	This study
RKCK5	F-, *Δ(araD-araB)*, *ΔlacZ*(::rrnB-3), Δ*tolA*::*gfp-tolA*, λ^-, *rph-1*, *Δ(rhaD-rhaB)*, *hsdR514*	This study
PB168	AB1157 *tolQ::tolQ-mCherry::kan*	(19)*
PB177	AB1157 *tolQ::tolQ-mCherry*	(19)*
PB199	AB1157 pBAD24/*FtsZ-GFP*	(44)*
DPD1718	*lac* Kan^r *lacZ*::*recAφluxCDABE*	(37)
