# Supplementary material for: Intermembrane crosstalk drives inner-membrane protein organization in Escherichia coli
Source: Nat Commun. 2018 Mar 14;9:1082. doi: 10.1038/s41467-018-03521-4 (PMC5852019; doi:10.1038/s41467-018-03521-4)
Supplement: Supplementary file 2 — Description of Additional Supplementary Files [file 41467_2018_3521_MOESM2_ESM.pdf]

Description of Additional Supplementary Files

File Name: Supplementary Movie 1
Description: 3D-SIM rotated image of an *E. coli* JW0729/pNP4, pRP5 cell expressing GFP-TolA (not induced) and BtuB (induced) showing the distribution of GFP-TolA in the IM.

File Name: Supplementary Movie 2
Description: 3D-SIM rotated image of an *E. coli* JW0729/pNP4, pRP5 cell expressing GFP-TolA (not induced) and BtuB (induced) showing clustered GFP-TolA fluorescence in the IM following binding of ColE9 to cells.

File Name: Supplementary Movie 3
Description: 3D-SIM rotated image of a dividing *E. coli* JW0729/pNP4, pRP5 cell expressing GFP-TolA (not induced) and BtuB (induced) showing a GFP-TolA septal ring at the cell division site.

File Name: Supplementary Movie 4
Description: 3D-SIM rotated image of a dividing *E. coli* JW0729/pNP4, pRP5 cell expressing GFP-TolA (not induced) and BtuB (induced) showing how binding of ColE9 to the OM sequesters GFP-TolA into clusters away from the division site.
